# Proximal aperture in *Cephalanthera longifolia* (L.) Fritsch (Orchidaceae) pollen: a rare germination site for angiosperms

**DOI:** 10.1186/s40529-024-00439-7

**Published:** 2024-11-14

**Authors:** Carola Purgina, Friðgeir Grímsson, Silvia Ulrich

**Affiliations:** 1 Department of Botany and Biodiversity Research, Division of Structural and Functional Botany, University of Vienna, Rennweg 14, Vienna, 1030 Austria; 2 Department of Historical Archaeology, Austrian Academy of Sciences (OeAW), Austrian Archaeological Institute (OeAI), Dominikanerbastei 16, Vienna, 1010 Austria

**Keywords:** Orchids, Pollen morphology, Pollen tetrad, Proximal pole, Ulcus

## Abstract

The pollen dispersal unit of the epidendroid species, *Cephalanthera longifolia*, is a soft pollinium consisting of loosely connected tetrads that are agglutinated by elastoviscin. With scanning electron microscopy, the reticulate exine is visible on the outer pollen grains of outer tetrads of a pollinium. The pollen grains are mostly arranged in planar-tetragonal tetrads or decussate tetrads and easily disintegrate into monads. Contrary to the inaperturate pollen in members of subfamily Epidendroideae known so far, *C. longifolia* exhibits ulcerate pollen. When pollen grains are attached in tetrads within a pollinium the apertures are obscured, as they are located on the proximal side of the pollen grains. The ulcus can only be observed when tetrads disintegrate, freeing the monads and exposing the proximal side of pollen grains for investigation by light and scanning electron microscopy. Proximal aperture configurations are rare among angiosperms and currently known only from few other species of flowering plants. This is the first report of an ulcerate proximal aperture within Orchidaceae.

## Background

Most pollen grains exhibit a resilient exine composed of biopolymer sporopollenin, that protects the male gametes from various environmental influences (Halbritter et al. 2018). However, this shielding wall is often interrupted by one or more apertures of distinct form, size, and position, that facilitate germination (e.g., Furness and Rudall 2004; Halbritter et al. 2018; Albert et al. 2022). Aperture patterns achieve a great diversity in angiosperm pollen (Ressayre et al. 2005) and differ both morphologically and ultrastructurally from the remaining pollen wall (e.g., Albert et al. 2022). In the aperture area the ektexine is often absent or reduced to granules, whereas endexine and intine layers increase in thickness and sometimes becomes bi- to multi-layered (Ressayre et al. 2005). The position of the aperture depends on different proteins, as recent studies have shown (e.g., Dobritsa and Reeder 2017; Albert et al. 2022). After meiotic cytokinesis, specific proteins accumulate at certain plasma membrane domains and are responsible for a close contact between plasma membrane and adjacent callose wall. This prevents deposition of primexine matrix and subsequently the formation of primexine (Dobritsa and Reeder 2017; Albert et al. 2022). The primexine is a layer that acts as template for exine formation (Gabarayeva et al. 2024). Still, it is not only proteins that are responsible for aperture formation, also the type of tetrad formation, the cytokinesis type, and the formation of a callose wall play vital roles when it comes to position and number of apertures (Albert et al. 2022). Despite the fact that apertures constitute a weak point in the pollen wall, they are important for a more rapid germination and play a role in the harmomegathic effect, as they often infold during desiccation and prevent the male gametes from drying out during pollination (Dafni and Ivri 1981; Halbritter and Hesse 2004; Albert et al. 2010b; Claessens and Kleynen 2013; Halbritter et al. 2018). In monocots the aperture is typically a distal sulcus (sulcate), ulcus (ulcerate), or the pollen grains are inaperturate (Furness and Rudall 2004; Albert et al. 2022). The aperture number, position, and type are related to the mode of meiotic cytokinesis (Blackmore and Crane 1998; Furness and Rudall 2004). In most of the eudicots a simultaneous cytokinesis leads to the typical tricolpate pollen type (Furness and Rudall 2004). In monocots the sulcate/ulcerate and inaperturate pollen types are associated with simultaneous or successive cytokinesis, and in basal angiosperms there are even intermediate types leading to an unstable aperture pattern and high variation (Furness and Rudall 2004).

For Orchidaceae, there are only two aperture types known so far, a distally placed sulcus and ulcus. Sulcate pollen is present in subfamily Apostasioideae. Ulcerate pollen has been documented in subfamily Cypripedioideae, in tribe Cranichideae of subfamily Orchidoideae, and in the tribes Tropidieae, Neottieae, and Diceratosteleae of subfamily Epidenroideae (formerly grouped together as Spiranthoideae) (Zavada 1990; Barone Lumaga et al. 2014; Chase et al. 2015). Orchids belonging to subfamilies Orchidoideae and Epidendroideae exhibit predominantly compact or sectile pollinia with mainly inaperturate pollen grains (Zavada 1990). The presence of a proximal positioned aperture (ulcus) in *Cephalanthera longifolia* (L.) Fritsch, belonging to the subfamily Epidendroideae, was first hinted by (Purgina et al. 2024b). Here we further investigate *C. longifolia* pollen and its unique aperture type using combined LM, SEM, and TEM, as well as tetrad stages and cytokinesis types during pollen/tetrad development. The proximal aperture placement observed in *C. longifolia* pollen is rare in angiosperms and has not been described for any other orchid species.

## Materials and methods

**Fig. 1:**
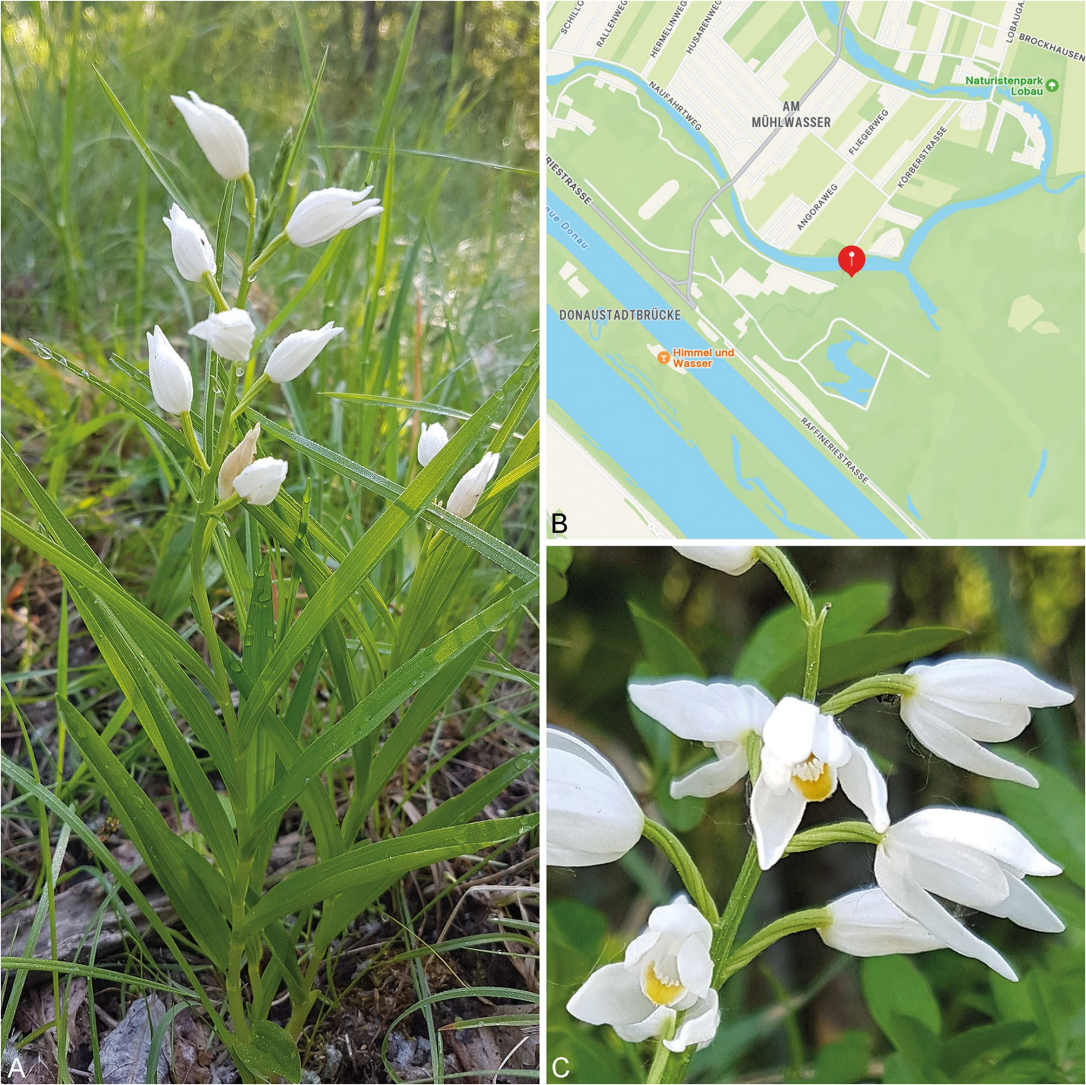
Macroscopic images of *Cephalanthera longifolia.* (**A**) Habitus. (**B**) Map showing the collection site of *C. longifolia* (red mark) in the national park Lobau. (**C**) Detail of the inflorescence

Pollen material of *C. longifolia* was collected from plants growing in the national park Lobau (Fig. 1A, B; GPS coordinates: 48.194859°, 16.476873°). Map showing the location where the plants were sampled can be accessed via the following link: https://gps-coordinates.org/my-location.php?lat=48.194859&lng=16.476873. Permission for collecting orchid plant material was granted by the MA 22. For plant identification the flora by Fischer et al. (2008) was used. For light microscopic (LM) investigations, pollinia from closed and open flowers (Fig. 1A, C) were investigated using various methods as described in Halbritter et al. (2018). Pollen material was: (1) placed in glycerin and hydrated in tap water for pollen shape and size, (2) stained with toluidine blue for pollen ornamentation and aperture condition, (3) stained with acetocarmine to detect cellular condition (bi- or trinucleate), and (4) acetolysed for pollen wall features (ornamentation, presence of sporopollenin). Measurements for polar axis and equatorial diameter (P/E-ratio) were conducted on ten individual pollen grains (monads) using LM micrographs as described in (Purgina et al. 2024b). For the pollen size, the length of the longest axis of hydrated pollen was also measured on ten individual grains following *PalDat* (2000 onwards, www.paldat.org). To detect tetrad stages, pollinia were extracted from flower buds at different flowering stages and from different inflorescences (Fig. 1A, C). All samples were investigated with an Olympus BX50 light microscope. Micrographs were taken with an integrated Olympus UC90 digital camera. For scanning electron microscopy (SEM) fresh pollinia were dehydrated with ethanol (70–85–96%) and acetone (100%) and critical point dried in an “Autosamdri^®^-815 - Series A tousimis Critical Point Dryer” (Halbritter et al. 2018). Air dried and critical point dried pollinia were then sputter coated with gold in a “BAL-TEC SCD 050 Sample Sputter Coater” and investigated with a JEOL JSM-IT300 Scanning Electron Microscope. Pollen and pollinia were examined in both dry and hydrated state, focusing on characteristics such as pollen ornamentation, shape, size, aperture configuration, and arrangement of pollen within tetrads of pollinia. For Transmission electron microscopy (TEM) pollen samples were stored in paper bags under cold storage conditions and primary fixed on the following day with 3% phosphate-buffered glutaraldehyde at room temperature for six hours. Post-fixation was prformed with 1% osmium tetroxide (OsO_4_) and 0.8% potassium ferrocyanide (K_4_[Fe(CN)_6_]) 2:1 for 15 h t 6 °C. After dehydration in a graded ethanol series (30–50%–70–85–96%, for 10 min each) and acetone (100%, two times for 10 min each), samples were infiltrated and embedded in an Agar low-viscosity resin (Halbritter et al. 2018). Ultra-thin sections of about 60–90 nm were cut using a Leica EM UC6 ultramicrotome and DiATOME Ultra 45° diamond knife. The sections were subsequently transferred onto formvar film-coated copper slit-grids. To test for the presence of an endexine, ultra-thin sections were stained with 1% potassium permanganate for five minutes and subsequently washed in drops of deionised water (Weber and Ulrich 2010). To test for the prsence of unsaturated lipids (lipid test), sections were stained with 0.2% thiocarbohydrazide (TCH) for 19 h and after washing stained with 1% silver proteinate (SP) for 30 min (Halbritter et al. 2018). The detection of polysccharides (Thiéry-test) follows the protocol by (Halbritter et al. 2018) and (Thiéry 1967) with a staining time of 19 h for 0.2% thiocarbohydrazide (TCH). Unstained and stained sections were investigated with a Zeiss EM900N Transmission Electron Microscope at 80 kV and documented with an Image SP-Program (ISPViewer64).

## Results

### Pollen description

**Fig. 2:**
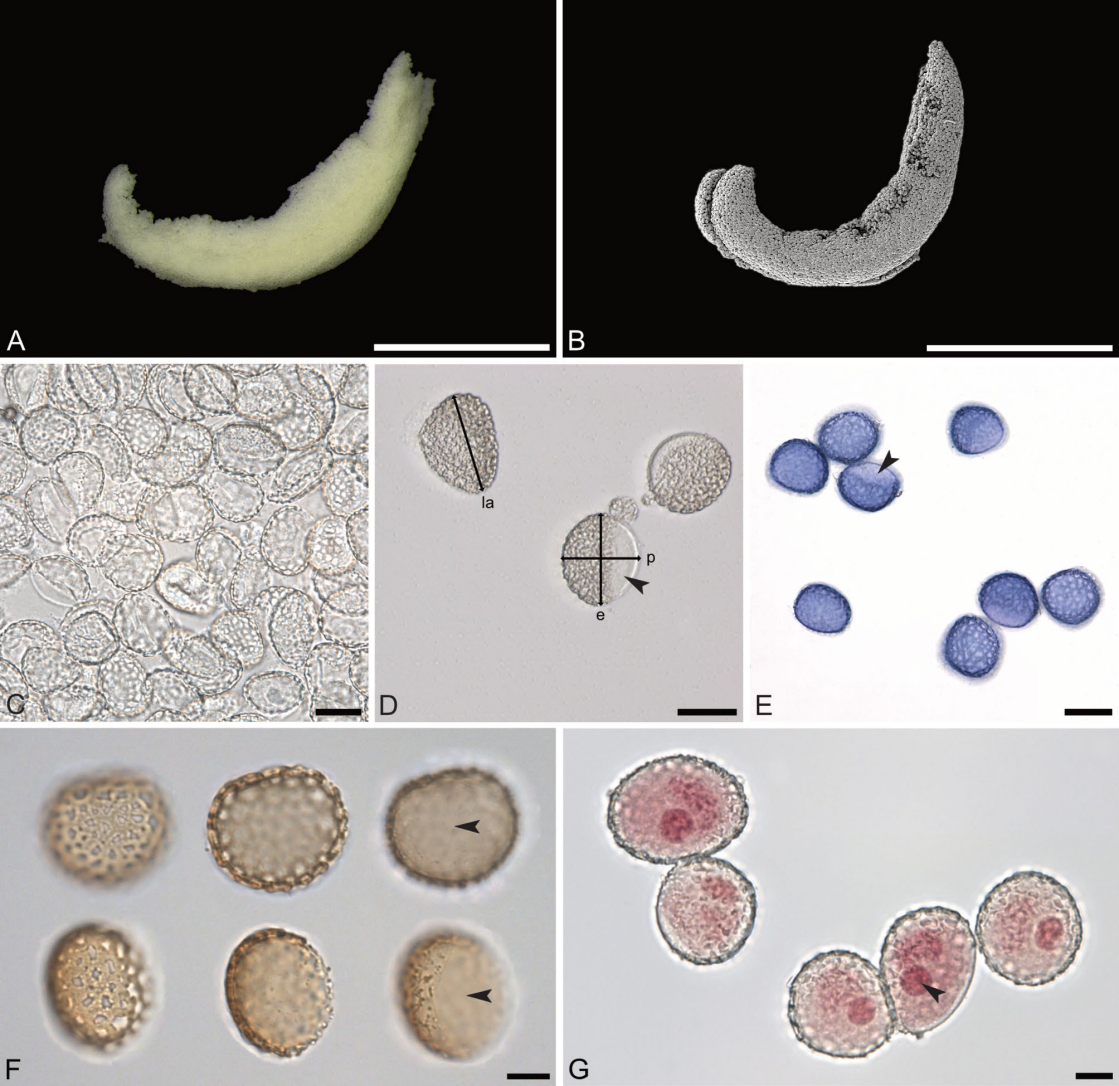
LM (**A**, **C**–**G**) and SEM (**B**) micrographs of *Cephalanthera longifolia* pollen. (**A**) Overview of pollinium. (**B**) Overview of pollinium (dry). (**C**) Tetrads disintegrating into monads; glycerine. (**D**) Monads hydrated in tap water; polar axis (p), equatorial diameter (e), longest axis (la), black arrowhead pointing to thin and smooth aperture membrane. (**E**) Monads hydrated and stained with toluidine blue, showing reticulate ornamentation (LM) and aperture area (black arrowhead). (**F**) Acetolyzed pollen grain in polar view (top row) and oblique equatorial view (lower row), with reticulate exine ornamentation and the not acetolysis-resistant aperture membrane (arrowheads), upper focus (left), optical cross section (middle), and lower focus (right); acetolyzed. (**G**) Pollen grains with a vegetative nucleus and a generative cell (black arrowhead); acetocarmine. Scale bars – 1 mm (**A**, **B**), 20 μm (**C**–**E**), 10 μm (**F**, **G**)

**Fig. 3:**
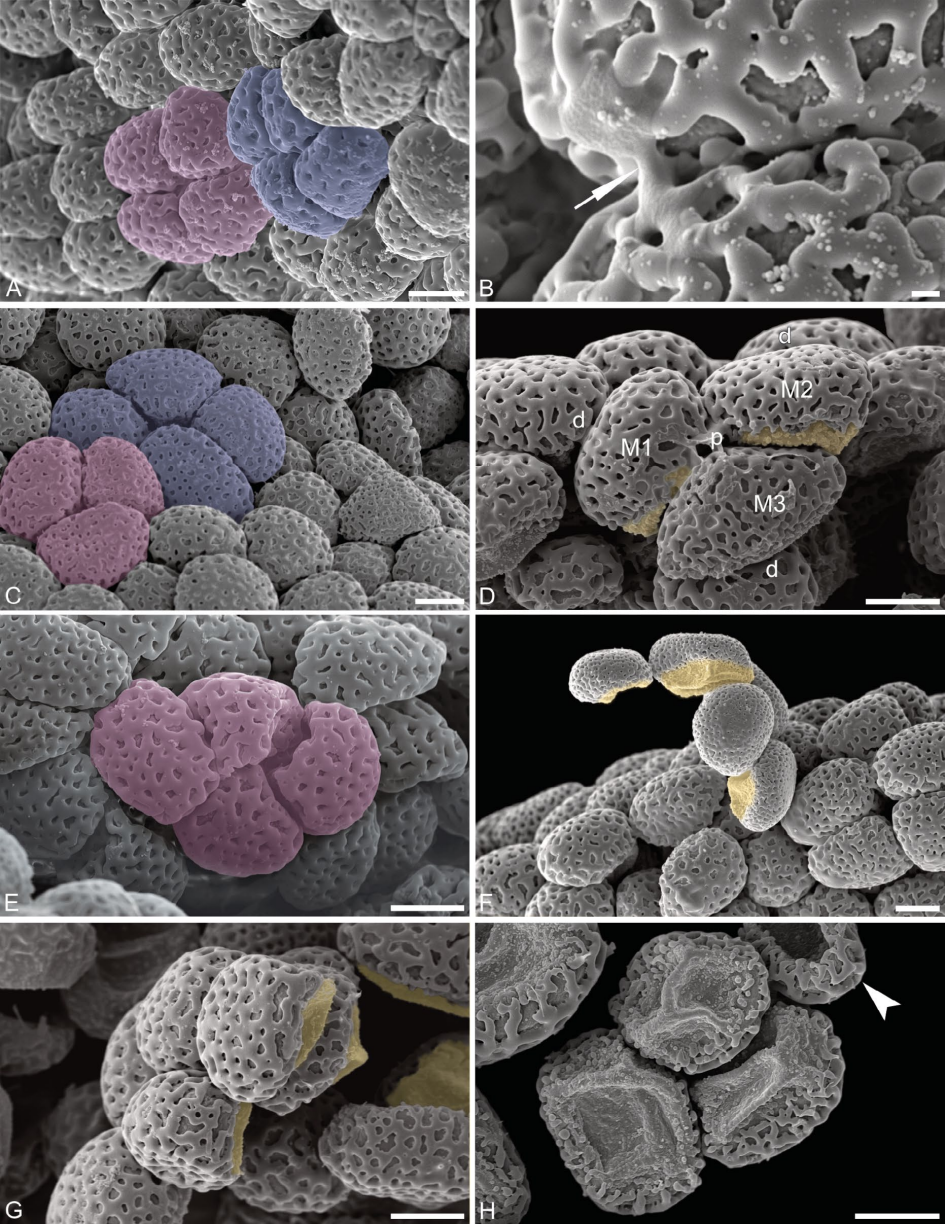
SEM micrographs of *Cephalanthera longifolia* pollen, (**A**, **B**, **D**–**H**, air dried; **C**, critical point dried). (**A**) Pollinium consisting of tetrads (light-pink, blue). (**B**) Pollen grains agglutinated by elastoviscin (white arrow). (**C**) Planar-tetragonal (blue) and decussate (light pink) tetrads. (**D**) Decussate tetrad showing ulci (yellow) in proximal position. (**E**) Planar-tetragonal tetrad (light-pink). (**F**) Disintegrating tetrad showing proximal ulci (yellow). (**G**) Individual pollen grains with ulcus in proximal position (yellow). (**H**) Cup-shaped (white arrowhead) and irregularly infolded pollen grains. Distal (d), individual pollen grains/monads (M1, M2, M3), proximal (p). Scale bars – 10 μm (**A**, **C**–**H**), 1 μm (**B**)

**Fig. 4:**
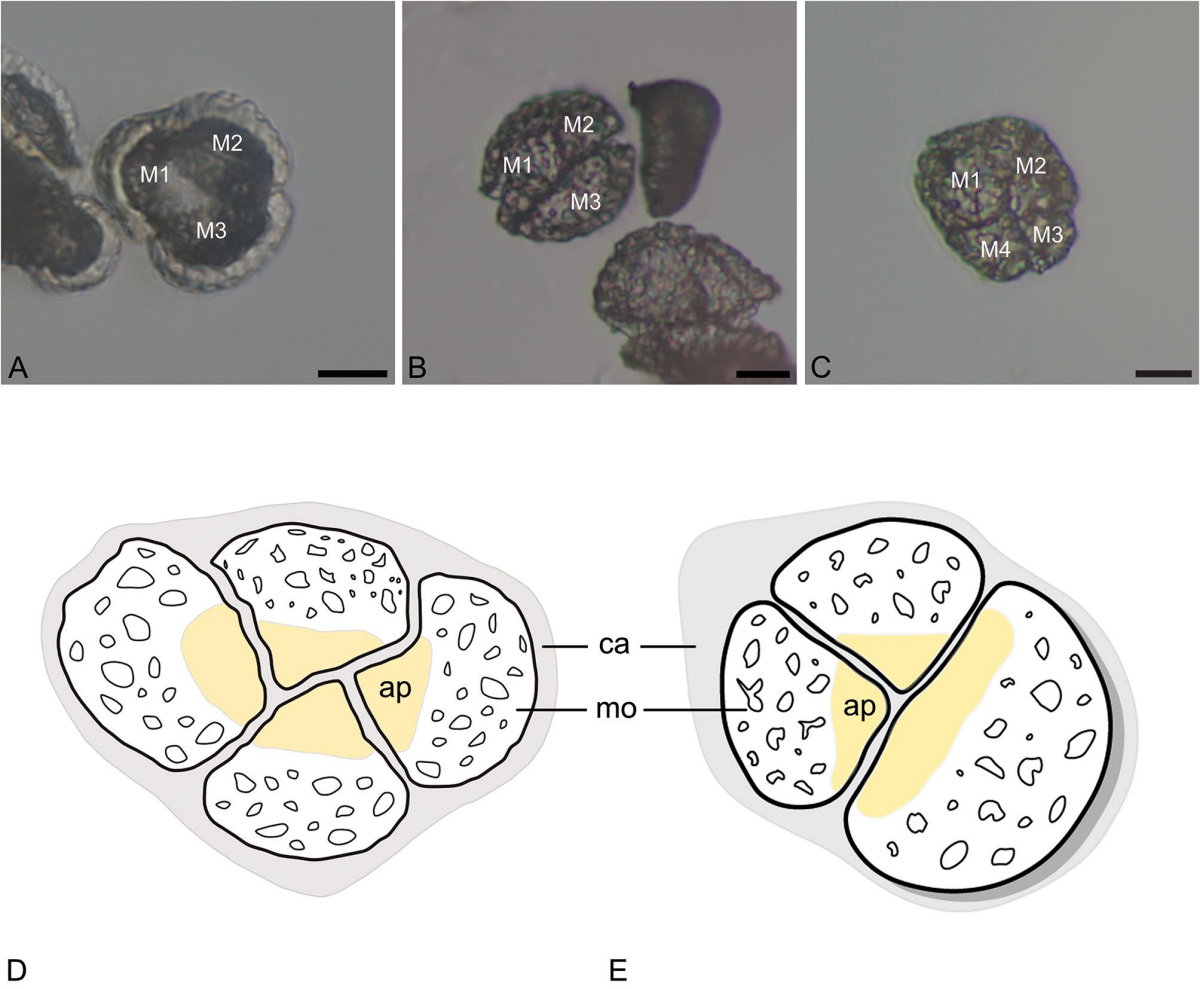
Tetrad types occurring in *Cephalanthera longifolia.* (**A**) Decussate tetrad (only three pollen grains visible). (**B**) Decussate tetrad. (**C**) Planar-tetragonal tetrad. (**D**) Planar-tetragonal tetrad, illustration. (**E**) Decussate tetrad, illustration. Individual pollen grains/monads (M1, M2, M3, M4), Aperture (ap), callose (ca.), monad (mo), Scale bars – 10 μm

**Fig. 5:**
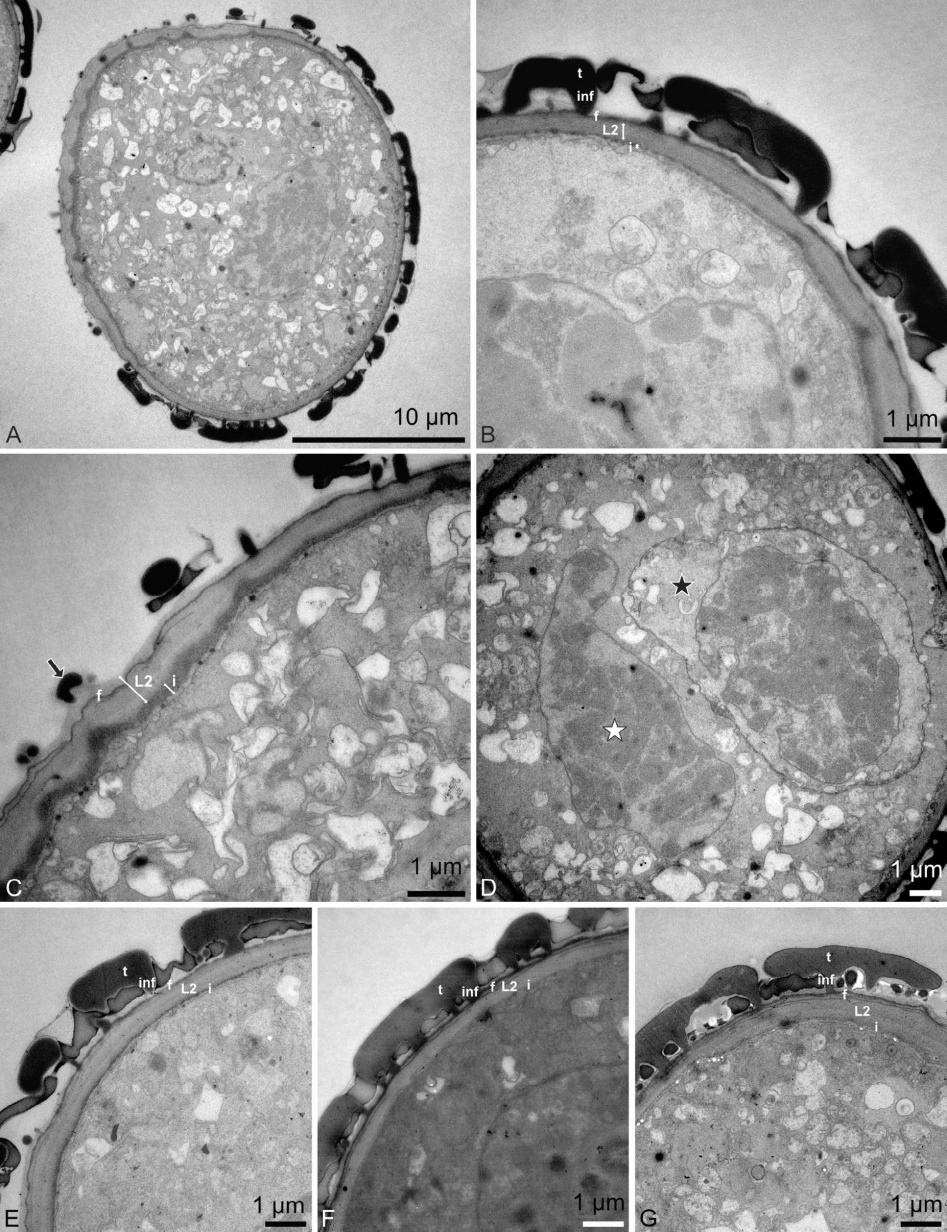
Transmission electron micrographs of *Cephalanthera longifolia* pollen. (**A**) Overview of monad with tectate-columellate pollen wall in the interapertural area, unstained. (**B**) Detail of pollen wall in the interapertural area, unstained. (**C**) Detail of pollen wall in the aperture area with loosely scattered granules (black arrow), unstained. (**D**) Pollen grain with vegetative nucleus (white star) and generative cell (black star), unstained. (**E**) Layer 2 (L2) electron-translucent with lipid test. (**F**) Layer 2 (L2) electron-translucent with KMnO_4_. (**G**) Layer 2 (L2) stained with Thiéry-test. Tectum (t), columellate infratectum (inf), foot layer (f), layer 2 (L2), intine (i). Scale bars – 10 μm (**A**), 1 μm (**B**–**G**)

The pollen dispersal unit of *C. longifolia* are soft, banana-shaped pollinia that are light yellow in color (LM; Fig. 2A, B). The pollinia consist of tetrads of various types, mainly planar-tetragonal and decussate, cohered by elastoviscin (SEM, LM; Figs. 3A–E and 4A–C). The tetrads are not permanent (LM; Fig. 2C–E) and disintegrate easily into monads (LM, SEM; Figs. 2C–G and 3F–H). The pollen units are the single pollen grains (monads), which are medium sized, with the longest axis of hydrated pollen between 26.4 and 33.1 μm in LM (Fig. 2F, G), the polar axis ranges from 22.5 to 26.7 μm, and the equatorial diameter ranges from 25.0 to 29.9 μm. The pollen grains are binucleate (LM, TEM; Figs. 2G and 5D), heteropolar, spheroidal to elliptic, and isodiametric to oblate (suboblate), with a P/E-ratio of 0.8–1.0 (LM; Fig. 2F, G). The pollen grains exhibit an acetolysis resistant reticulate pollen wall, which transitions into a thinner and smoother aperture membrane, that is not resistant to acetolysis (LM; Fig. 2F, G). The ornamentation in interapertural areas is foveolate to reticulate, and heterobrochate with robust and thick muri (LM, SEM; Figs. 2F and 3). The aperture membrane is ornamented with nanoverrucae, microverrucae, and granula (SEM; Fig. 3H). The aperture is an ulcus (LM; Fig. 2F, G), that in dehydrated pollen grains looks cup-shaped or irregularly infolded, often resembling a trilete impression mark (SEM; Fig. 3H). The position of ulci in pollen that remain in tetrads is proximal (SEM, LM; Figs. 3A and C–G and 4A–C). The reduction of exine towards apertures is also evident in TEM, where the tectum and infratectum are reduced to granules and layer 2 (of unclear chemical nature), and the intine becomes thicker (TEM; Fig. 5A, C). The interapertural areas show a structured sporopollenin tectate-columellate wall (TEM; Fig. 5A, B) consisting of a semitectum, a columellate infratectum, a thin-continuous foot layer, a subjacent layer 2, and a thin intine (TEM; Fig. 5B). Staining with the lipid test or potassium permanganate did not indicate a lipidic nature for layer 2 (TEM; Fig. 5E, F) and excludes it as an endexine. Therefore, layer 2 is assumed to be of polysaccharide nature and represents a second intine layer, meaning *C. longifolia* has a bilayered intine (TEM; Fig. 5G).

### Proximal aperture condition in *C. longifolia* pollen

Tetrads of *C. longifolia* are not permanent and disintegrate into monads when transferred into drops of water or glycerine (LM; Figs. 2C–G and 4A–C). In complete tetrads observed with SEM, the aperture was not visible, indicating its proximal position. This was also documented for pollen that were parts of outermost tetrads in complete or broken pollinia (SEM; Fig. 3F–H). To further confirm the proximal position of the aperture (ulcus), pollinia at different developmental stages from both open and closed flowers (Fig. 1A, C) were investigated in an attempt to discover young tetrad stages (LM; Fig. 4A–C). Unfortunately, most of the tetrads observed had already disintegrated into monads in the investigated pollinia. Therefore, an early tetrad stage, with the tetrads still enclosed in callose, was not observable. Still, tetrads at later stages of development, released from callose were observed, (LM; Fig. 4A–C), both complete (LM; Fig. 4B, C) and incomplete. In LM, the proximal aperture is hidden at tetrad stage (Fig. 4D, E), and can only be observed in incomplete tetrads or when they have disintegrated (LM, Fig. 4A). The tetrad types observed in *C. longifolia* were either planar-tetragonal (Fig. 4D) or decussate (Fig. 4E). Due to observations of tetrad arrangement in LM and SEM, it was possible to localize the apertures on monads still arranged in different tetrad types. Based on these observations, the main tetrad types of *C. longifolia* were schematically illustrated (Fig. 4) for a better visualization of the proximal ulcus.

## Discussion

Based on our detailed investigations using combined LM, SEM, and TEM we show that the ulcus of *C. longifolia* is located on the proximal pole of pollen grains. This is the first documented case of a proximal aperture in orchids. Barone Lumaga et al. (2014) also described an ulcerate aperture for *C. longifolia* but assumed a distal position, as an ulcus is usually located distally (e.g., Halbritter et al. 2018). As the tetrads in *C. longifolia* are not permanent and easily disintegrate, the interpretation of the aperture condition in orchids with monads as pollen units remains a challenge and must be clarified by investigating early and late tetrad stages. Based on our observations of *C. longifolia* pollen within complete and incomplete tetrads in LM and SEM, we demonstrate that the ulcus of *C. longifolia* is located on the proximal pole of the pollen grain.

The microsporogenesis of orchid pollen is often described as simultaneous (e.g., Blackman and Yeung 1983; Kant and Bhanwra 2010; Kant 2019). Depending on the cytokinesis type differently constructed tetrads are formed (Purgina et al. 2024b). Tetragonal, T-shaped, decussate, Z-shaped, and linear tetrads originate from successive cytokinesis, whereas tetrads resulting from simultaneous cytokinesis are either tetrahedral, rhomboidal, tetragonal, or decussate (Albert et al. 2011; Purgina et al. 2024a, b). Previous studies by Purgina et al. (2024a, b) on orchid pollen showed that in subfamilies Epidendroideae and Orchidoideae both successive and simultaneous cytokinesis types, as well as intermediate types, can occur within single species. Examples for tetrad types resulting from either successive or simultaneous cytokinesis are found in the epidendroid species *Bulbophyllum retusiusculum*, *Oncidium crocidipterum*, and *Polystachya cultriformis*, whereas in *Dendrobium* x *delicatum* both types co-occur. Purgina et al. (2024a) also demonstrated that the cytokinesis process in a single taxon can be both simultaneous and successive, as documented for *Anacamptis* (*A. coriophora* and *A. morio*). Other taxa reported exhibited either simultaneous (*Pteroglossa roseoalba*, *Orchis militaris*) or successive (*Neotinea ustulata*, *Ophrys sphegodes*) cytokinesis cell division based on their tetrad types (Purgina et al. 2024a, b). In case of *C. longifolia* differently constructed tetrads of both cytokinesis types are present within the same pollinium. This means that *C. longifolia* produces either planar-tetragonal tetrads, and/or decussate tetrads resulting from either simultaneous and/or successive cytokinesis. Such a phenomenon may also be present in *Asimina triloba* L. (Annonacaeae), that produces pollen with a proximal sulcus and monads arranged in permanent planar-tetragonal and decussate tetrads (Hesse et al. 2009; Heigl and Halbritter 2024).

Over time the position of apertures in spores and pollen has changed several times during the evolution of land plants (Klaus 1987). While the aperture in spores was/is exclusively proximally located, it has shifted towards the distal pole in gymnosperms and primitive angiosperms. A sulcus in distal position (anasulcus) is described as the most common aperture type among monocots and the ranalean complex, which comprises the basal groups of flowering plants, and is consequently considered the most ancestral aperture configuration among angiosperms (Walker 1974; Walker and Doyle 1975; Zavada 1983). The aperture condition in angiosperms, however, is much more diverse, which can be attributed to multiple transitions over time (Walker 1974). In advanced angiosperms the aperture(s) subsequently shifted towards the equator or is/are globally situated (Klaus 1987). However, there are also deviations from this trend, like the reversal to proximal aperture position as evident from only few angiosperms. Six species/genera have been documented within the dicots: (1) *Asimina triloba* L. Dunal, Annonaceae, Magnoliales (Hesse et al. 2009), (2) *Pseudoxandra williamsii* (R.E.Fr.) R.E.Fr., Annonaceae, Magnoliales (Doyle and Le Thomas 1994), (3) *Annona cherimola* Mill., Annonaceae, Magnoliales (Rosell et al. 1999), (4) *Cuphea* P.Browne, Lythraceae, Myrtales (Albert et al. 2010a), (5) *Drosera capensis* L., Droseraceae, Caryophyllales (Takahashi 1988), and (6) *Dionaea* J.Ellis, Droseraceae, Caryophyllales (Halbritter et al. 2012). Within monocots five species/genera have been reported producing pollen with a proximal aperture: (1) *Tofieldia* Huds., Tofieldiaceae, Alismatales (Huynh 1976; Albert et al. 2010a), (2) *Beschorneria yuccoides* K.Koch, Asparagaceae, Asparagales, (Hesse et al. 2009), (3) *Vanilla* Plum. ex Mill., Orchidaceae, Asparagales (Albert et al. 2010a), (4) *Tulipa* L., Liliaceae, Liliales (Albert et al. 2010a; Halbritter 2016; Halbritter and Buchner 2016), (5) *Tinantia* Scheidw., Commelinaceae, Commelinales (Harley 2004; Hesse et al. 2009) and (6) *Tillandsia leiboldiana* Schltdl., Bromeliaceae, Poales (Albert et al. 2010a). Of these taxa, in which a shift of the aperture from distal to proximal pole was found, some must be excluded after critical review. For example, the proximal sulcus reported for *Cuphea lobelioides* Griseb. could not be confirmed, as only syncolpate pollen is documented for this species (Graham and Graham 1968). Also, Graham and Graham (1968) recognized a high variability in pollen morphology within species of the eurypalynous genus *Cuphea*, but not a single species with proximal aperture. As the aperture condition varies from diporate to tricolporate to syncolpate, and with apertures fused at the poles, this might have been a misinterpretation. In case of the monocot genus *Tofieldia*, pollen is usually disulcate or sulcate (Huynh 1976; Albert et al. 2010a). In the study by Huynh (1976), *T. calyculata* L. is described as disulcate, with one sulcus on the proximal pole, but in this case, there is an additional aperture but no switch from distal to proximal. In the genus *Tulipa*, pollen is typically distally sulcate, often in combination with an operculum, but only in few cases with additional wall thinning (tenuitates) on the proximal pole, as documented for *T. kaufmanniana* Regel (Halbritter 2016) and *T. batalinii* Regel (Halbritter and Buchner 2016), but there is a lack of studies on their functional aspects. The proximal aperture in pollen of *Vanilla*,* Pseudoxandra*, and *Tinantia* mentioned by Albert et al. (2010a) could not be confirmed, as there is no available literature showing examples of proximal apertures for these genera. In the few studies on *Vanilla* pollen, the grains are either inaperturate (*V. planifolia* Andrews, Halbritter and Svojtka 2016a) or porate with a variable number of apertures ranging from 3 pori (*V. aphylla* Blume, Halbritter and Svojtka 2016b) to more than 6 pori (*V. pompona* Schiede, Halbritter and Svojtka 2016c). In the latter case the pori are situated on one half of the heteropolar pollen, but without tetrad observations it remains unclear if they are on the proximal or distal half.

Proximal apertures documented so far are sulcate or ulcerate and located on the proximal half of pollen grains or directly at the proximal pole (Hesse et al. 2009), such as documented for *Beschorneria yuccoides* and the Annonaceae (*Annona cherimola* and *Asimina triloba*; Hesse et al. 2009; Halbritter et al. 2018). In the annonaceous genus *Pseudoxandra* R.E.Fr., for example, a shift from distally sulcate (anasulcuate) to proximally sulcate or ulcerate (catasulcate-cataulcerate) is observable, including transition states with a reduced distal aperture and a proximal thinning (Walker 1974; Doyle and Le Thomas 1994). In the genus *Annona*, another transition from proximally sulcate or ulcerate (catasulcate-cataulcerate) to inaperturate is noticeable (Walker 1971a, b, 1974; Hesse et al. 2009).

Such aperture transitions are also evident in Orchidaceae, where the most advanced tribes lost the sulcate aperture condition, an evolutionary trend also documented for several other monocots (Zavada 1983). The basal orchid subfamilies, Apostasioideae and Cypripedioideae, exhibit monosulcate pollen (dispersed as monads). In the intermediate subfamily Orchidoideae and the lower epidendroid tribe Neottieae, monosulcate and ulcerate pollen occurs (dispersed mostly as tetrads/pollinia/pollinaria). The higher epidendroids have inaperturate pollen (dispersed as pollinia/pollinaria) (Zavada 1983; Li et al. 2019).

A proximal thinning, which is intended to function as germination site, was also described for few other species within the subfamily Epidendroideae, such as *Didymoplexis pallens* Griff., *Epipogium aphyllum* Sw., and *Arethusa bulbosa* L. (Hesse et al. 1989). However, the authors did not find a clear or defined aperture area on the monads with SEM, but a proximal thinning was visible in the ultrastructure formed by a reduction of the ektexine and thinning of the intine towards the proximal half of the monads (Hesse et al. 1989). Such a pollen wall reduction was also observed in previous studies by Purgina et al. (2024b) on epidendroids, where pollinia are described having tetrads with inaperturate monads, a feature that seems to be characteristic for the epidendroids. Hence, the structural thinning cannot be considered a sulcus nor a typical aperture, since apertures are described as *“a region of the pollen wall that differs significantly from its surroundings in morphology and/or anatomy […]”* (Hesse et al. 1989; Halbritter et al. 2018, p. 440).

Based on this, the aperture condition of *C. longifolia* (Tribe Neottieae, subfamily Epidendroideae) corresponds to the before mentioned aperture transition in orchids over time, except that the ulcus is not distal but proximal. Why such reversals from a distally to a proximally located aperture occurs and whether this is advantageous for pollen viability or germination, is not yet explained (Albert et al. 2010a). In case of *C. longifolia*, an advantage in case of germination is unlikely, as pollen grains do not remain in tetrads but disintegrate into monads. An advantage in pollen viability is more likely, since the ulcus extends over half of the pollen grain and, due to the thinner wall, represents a weak point concerning desiccation. With the ulcus located proximally within tetrads it is more protected than the distal pollen half that is directed outwards and is more exposed to environmental influences. This may be advantageous for pollen viability in terms of temporary desiccation.

The proximal ulcerate aperture condition documented for *C. longifolia* has not been described for any other orchid species so far and is unique among Orchidaceae. To establish whether this unique aperture condition is more frequent among orchids having monads as pollen units and if this aperture condition is restricted to certain subfamilies of Orchidaceae, further studies are warranted.

## Conclusions

Our study unveiled a unique aperture condition for *C. longifolia* pollen, which exhibit an ulcus in proximal instead of distal position. This is the first documented case of a proximal aperture in orchids, which was discovered by a combined study using LM, SEM, and TEM. This raises the question whether proximal aperture conditions are more common among orchids or if *C. longifolia* is an exception. Even though the ulcus in *C. longifolia* pollen is extending over half of the grain, its proximal position and placement within the tetrad/pollinium may provide an advantage in pollen viability and protect the pollen against desiccation. An advantage in case of germination is unlikely, as pollen grains do not remain in tetrads but disintegrate into monads. The fact that planar-tetragonal and decussate tetrads co-occur in the same pollinium of *C. longifolia*, indicates that both cytokinesis types, simultaneous and/or successive, are involved, leading to an unstable aperture pattern and thus to a high degree of variation. This demonstrates the importance of a comprehensive palynological study including pollen developmental stages, tetrad types, and aperture configurations. Furthermore, this shows the complexity and variability of the formation and position of pollen apertures even within a family and/or subfamily. This also indicates that mechanisms involved are more complex than previously thought and there is always an exception to every rule. To determine whether this unique aperture condition is present in other orchids and/or if it is restricted to specific taxa/tribes/subfamilies, more comprehensive studies on additional orchid species are needed.

## Data Availability

All data supporting the findings of this study are either part of the published manuscript or available from C.P. upon reasonable request.
